# Editorial: Innate immunity in vasculitis

**DOI:** 10.3389/fimmu.2024.1432069

**Published:** 2024-06-03

**Authors:** Alexandre Wagner Silva De Souza, Joshua Daniel Ooi, Durga Prasanna Misra, Yong Zhong, Kelly L. Brown

**Affiliations:** ^1^ Rheumatology Division, Department of Medicine, Universidade Federal de São Paulo – Escola Paulista de Medicina, São Paulo, SP, Brazil; ^2^ Centre for Inflammatory Diseases, Department of Medicine, Monash Medical Centre, School of Clinical Sciences, Monash University, Clayton, VIC, Australia; ^3^ Department of Clinical Immunology and Rheumatology, Sanjay Gandhi Postgraduate Institute of Medical Sciences, Lucknow, Uttar Pradesh, India; ^4^ Department of Nephrology, Xiangya Hospital, Central South University, Changsha, China; ^5^ British Columbia (BC) Children’s Hospital Research Institute, Centre for Blood Research, and Department of Pediatrics, University of British Columbia, Vancouver, BC, Canada

**Keywords:** systemic vasculitis, innate immunity, monocytes, macrophages, neutrophils, cytokines

Primary systemic vasculitis is a heterogeneous group of rare disorders characterized by inflammation and/or necrosis affecting the blood vessel wall as the primary target of the immune system (1). Blood vessels of different types and sizes may be affected by the inflammatory process that, in turn, may involve several organs and organ systems in multiple combinations (2). Deriving from the 2013 Chapel Hill Consensus Conference, primary systemic vasculitides are classified as large-vessel, medium-vessel, small-vessel and variable-vessel vasculitis based on the size of the blood vessels that are predominantly affected by the inflammatory process (3).

Although systemic vasculitides are commonly considered autoimmune in nature owing to the presence of autoreactive antibodies in the majority of patients, the innate immune system also has an important role in the pathogenesis of systemic vasculitides. Innate immune cells including neutrophils, monocytes, macrophages, NK cells, dendritic cells, eosinophil, and γδ T cells are found in inflammatory infiltrates in affected vessels and act as effectors driving inflammation and damage to vessel walls (4). A detailed understanding of innate immunity mechanisms that contribute to inflammation and damage in systemic vasculitis, however, is still lacking.

In this Research Topic, Tao et al. developed a NETosis score model and identified six NETosis-related genes with potential predictive utility in antineutrophil cytoplasmic antibody (ANCA)-associated glomerulonephritis (ANCA-GN). The expression of NETosis-related genes had a significant positive correlation with particular immune processes in ANCA-GN involving chemokines (CCR), macrophages, T-cell inhibition and tumor-infiltrating lymphocytes, as well as an inverse correlation with kidney function. Regarding IgA vasculitis (IgAV), Qin et al. performed a bidirectional Mendelian randomization study to analyze the interaction between IgAV and different inflammatory factors including C-reactive protein (CRP), growth factors, chemokines, and cytokines. Higher CRP and interleukin (IL)-8 levels were associated with an increased risk of IgAV, whereas genetically predicted IgAV was associated with decreased levels of TNF-β. In Kawasaki disease (KD), Uittenbogaard et al. described an association between polymorphisms in the FCGR2/3 locus and an increased susceptibility to KD, but not, however, to intravenous immunoglobulin (IVIG) resistance or the development of coronary artery aneurysms. Finally, Ishikawa et al. showed that in Takayasu arteritis (TAK) patients, anti-integrin ανβ6 antibodies were present more frequently in individuals with TAK-associated ulcerative colitis (UC) compared to TAK patients without UC. In addition, no association was observed between anti-integrin ανβ6 antibodies and the HLA-B*52 carrier status in TAK patients without UC.

In conclusion, the articles published in this Research Topic investigated genetic susceptibility factors for the development of IgAV and KD, explored the relationship between anti-integrin ανβ6 antibody seropositivity and ulcerative colitis manifestations in TAK, and, finally, evaluated NETosis-associated genes in glomerulonephritis of ANCA-associated vasculitis. We hope this Research Topic will bring some new insights into the pathogenesis of systemic vasculitis and will encourage the development of research projects to further unravel the role(s) of innate immunity in vasculitis.

## Author contributions

AS: Writing – original draft. JO: Writing – review & editing. DM: Writing – review & editing. YZ: Writing – review & editing. KB: Writing – review & editing.
